# Transfusion rates in oral and maxillofacial surgery and their influencing factors in the context of patient blood management

**DOI:** 10.1007/s00784-026-06902-y

**Published:** 2026-05-13

**Authors:** Nils Mönnikes, Jakob Fenske, Claudius Steffen, Vassilisa Wu, Sascha Treskatsch, Max Heiland, Carsten Rendenbach, Axel Pruß

**Affiliations:** 1 Department of Oral and Maxillofacial Surgery, Charité - Universitätsmedizin Berlin, corporate member of Freie Universität and Humboldt Universität zu Berlin, Augustenburger Platz 1, 13353 Berlin, Germany; 2 Institute of Transfusion Medicine, Charité - Universitätsmedizin Berlin, corporate member of Freie Universität and Humboldt Universität zu Berlin, Charitéplatz 1, 10117 Berlin, Germany; 3 Department of Anesthesiology and Intensive Care Medicine, Campus Benjamin Franklin, Charité - Universitätsmedizin Berlin, corporate member of Freie Universität and Humboldt Universität zu Berlin, Hindenburgdamm 30, 12200 Berlin, Germany

**Keywords:** Anemia, Transfusion rate, Patient blood management, Blood transfusion, Free flap reconstruction, Oral and maxillofacial surgery

## Abstract

**Objectives:**

Existing evidence on transfusion requirements in oral and maxillofacial surgery (OMFS) is limited to selected indications. This study aimed to provide an overview of transfusion rates across the full diagnostic spectrum and to identify factors influencing transfusion rates with relevance for patient blood management (PBM).

**Materials and methods:**

All operated OMFS patients from a five-year period (*n* = 13,239) were retrospectively analyzed. Diagnosis-specific transfusion rates were determined, followed by a subgroup analysis of free flap surgeries. Logistic regressions identified factors influencing transfusion rates. ROC analysis in the free flap subgroup determined preoperative hemoglobin cut-off values for increased transfusion risk. Differences in treatment course associated with preoperative anemia were assessed.

**Results:**

Overall transfusion rate was 5.1%. Microvascular free flap surgery was the primary driver of transfusion with a rate of 58.8%, independent of underlying pathologies. Non-oncologic indications requiring free flap reconstruction showed high transfusion rates similar to oncologic indications, whereas the same diagnoses without free flaps had rates < 5%. Free flap reconstruction (OR 5.21) and preoperative anemia (OR 6.25) were the strongest factors influencing transfusion rates. ROC analysis identified preoperative hemoglobin of 12.25 g/dl as risk threshold for intraoperative transfusion. Preoperative anemia was associated with a less favorable course regarding intensive care unit treatment, in-hospital mortality and hospital length of stay.

**Conclusions:**

Transfusion rates in OMFS are generally low but increased in reconstructive free flap surgery.

**Clinical relevance:**

These findings offer an evidence base for targeted PBM strategies, including early identification and treatment of preoperative anemia, like intravenous iron therapy in free flap patients, and transfusion rate-adapted blood product preparation to improve perioperative management.

**Supplementary Information:**

The online version contains supplementary material available at 10.1007/s00784-026-06902-y.

## Introduction

Surgeries in general, as well as specifically in oral and maxillofacial surgery (OMFS), may be associated with the need for allogeneic blood transfusions [1]. The transfusion probability of elective surgery can be described by transfusion rates. The transfusion rate is defined as the number of transfusions for a diagnosis or surgery in relation to the total number of this diagnosis or surgery in a specific period. The differentiated recording of transfusion rates and their influencing factors is relevant for optimal therapy of surgical patients. Increased transfusion rates (in Germany > 10%) require extended information about allogeneic blood transfusions - including autologous hemotherapy procedures - as well as the risk of blood loss, the initiation of extended blood group serology diagnostics and, in addition, the preoperative preparation of allogeneic blood products in sufficient quantities [2]. These implications are of utmost importance to improve preoperative planning and therefore affect the entire area of patient blood management (PBM) [2, 3].

The need for perioperative blood transfusions has been described for head and neck cancer in the field of OMFS, such as oral squamous cell carcinoma (OSCC) and the reconstruction with microvascular free flaps [4–9]. Further diagnoses and procedures in OMFS for which transfusion rates are known include orthognathic surgery [10, 11] and the treatment of patients with craniofacial deformities [12, 13].

However, the number of studies dealing with transfusion rates in OMFS and their influencing factors remains limited. Although existing studies deal with selected pathologies and surgical interventions, the study populations are small in size and the described transfusion rates vary between the different studies [7, 9, 14, 15].

The aim of this study was to provide a differentiated overview of transfusion rates across the full diagnostic spectrum of OMFS and to identify factors influencing transfusion rates. We assume that the results may provide an evidence base for PBM in OMFS, support risk-adapted transfusion strategies and contribute to improved perioperative management.

## Methods

### Study design

A retrospective cohort study was designed and conducted. The retrospective patient data were collected as part of standard hospital care within the specialties of OMFS and transfusion medicine.

The study was approved by the Ethics Committee of Charité - Universitätsmedizin Berlin (EA2/057/23) in accordance with the Declaration of Helsinki including a data protection statement. The study was conducted at the Department of Oral and Maxillofacial Surgery at Charité - Universitätsmedizin Berlin in cooperation with the Institute of Transfusion Medicine at Charité.

### Study population & data research

To determine transfusion rates, all patient cases with an OMFS diagnosis at the Department of Oral and Maxillofacial Surgery at Charité - Universitätsmedizin Berlin were initially recorded with and without a transfusion in the defined observation period. In a five-year observation period, patients were included who were admitted on April 1st 2017, at the earliest and discharged on April 30th 2022, at the latest. The prerequisite for inclusion was an OMFS diagnosis assigned to the individual patient in the hospital’s internal controlling system and coded according to the "International Statistical Classification of Diseases and Related Health Problems" (ICD-10-GM Version 2024) [16]. Subsequently, all patients who did not undergo surgery or who underwent outpatient surgery were excluded from this cohort, so that only all inpatients and patients coded according to the “Operations and Procedures Code” (OPS Version 2024) [17] were analyzed during this period. Patients of all sexes and ages including minors (age < 18 years) who met the study criteria were included. Patients were excluded if there was insufficient documentation of transfusion, diagnosis and surgery or if other inclusion criteria were missing.

The information on transfused blood products was provided via the hospital’s blood bank system and compared with the data stored in the hospital’s controlling system. This data was supplemented by laboratory parameters from the hospital’s database. Data from operation protocols and occupancy lists were also recorded. Both patients who were admitted to a general ward and those who were temporarily receiving intensive care unit treatment and transfusions were considered. To address the aim of the study, age, sex, degree of obesity according to World Health Organization (WHO), pre- and postoperative hemoglobin as last recorded value before the operation and first value after the operation, red blood cell (RBC) units, platelet units, fresh frozen plasma (FFP) units, surgery duration, diagnosis, intensive care unit (ICU) treatment, hospital length-of-stay (H-LOS) and in-hospital mortality were recorded. Transfusions were recorded for the overall time of hospitalization and additionally analyzed according to the time point of transfusion, differentiating between preoperative, intraoperative and postoperative transfusions, defined as follows: preoperative (from hospital admission until surgery), intraoperative (during surgery), and postoperative (from surgery until discharge). The focus of the laboratory blood parameters with possible influence on the transfusion rate was hemoglobin as the most important parameter in anemia diagnostics (anemia: hemoglobin < 13.5 g/dl for men, < 12.0 g/dl for women according to the standard values of the hospital laboratory) and transfusion trigger. Due to the retrospective study design, complete laboratory data were not available for all patients with regard to hemoglobin values. Patients were not excluded from the analysis if hemoglobin values were missing, in order to preserve the representativeness of the full OMFS cohort. Hemoglobin values were analyzed for patients with available laboratory documentation in the hospital database. No imputation of missing laboratory values was performed. After initial screening of data, ICD codes were grouped by content to determine diagnosis-related transfusion rates, following the terminology and higher-level diagnostic categories defined in the ICD-10-GM classification. The groups of diagnoses analyzed were “malignant neoplasms”, “benign neoplasms, “diseases of the oral cavity, salivary glands and jaws”, “diseases of the respiratory system”, “diseases of the skin and subcutaneous tissue”, “diseases of the musculoskeletal system and connective tissue”, “congenital malformations of eye, ear, face and neck”, “trauma”, “complications of surgical and medical treatment” and “other OMFS diagnoses”, the latter representing a composite group of less frequent ICD-10-GM diagnoses not assignable to the aforementioned categories. For diagnoses with increased transfusion rates (> 10%), the associated surgical interventions were analyzed to determine procedure-related transfusion rates. Those were predominantly microvascular free flaps from different donor regions for major reconstructive OMFS surgery.

### Statistical analysis

The statistical analyses were performed with RStudio (version 4.3.1, R Project for Statistical Computing). The transfusion rate in percent is defined as the number of transfusions for a specific diagnosis or surgery divided by the total number of this diagnosis or surgery during a set time. The mean value and standard deviation (SD) were calculated for metrically scaled variables. Absolute and relative frequencies were calculated for nominally or ordinally scaled variables. 95% confidence intervals (CI) were calculated for all relevant parameters. To test two groups for differences in the parameters under consideration, t-tests (in the presence of normal distribution) or Wilcoxon tests were used for metrically scaled variables. In case of more than two groups, Kruskal-Wallis tests were used. For nominally scaled variables, Fisher’s exact test or chi-square test were used. A significance level of 0.05 (α = 5%) was selected for the statistical test procedures used. Univariate and multivariate logistic regression analyses were carried out to check whether there were significant influencing factors on the transfusion rate. Odds ratios (OR) including 95% CI were calculated.

Receiver operating characteristic (ROC) analysis was performed to identify a threshold of preoperative hemoglobin levels for overall and for intraoperative transfusion risk in patients undergoing reconstructive free flap surgery. The area under the curve (AUC) was calculated as a measure of discrimination. Hemoglobin cut-off values associated with an increased transfusion risk were determined using the Youden index. Sensitivity, specificity, positive predictive value (PPV), and negative predictive value (NPV) were analyzed for the calculated thresholds.

## Results

The initial study population comprised 697 transfused and 14,554 non-transfused OMFS patients. After applying the inclusion and exclusion criteria, 669 transfused and 12,570 non-transfused patients with the same diagnoses in the same period remained in the total analyzed population of 13,239 patients.

A total of 3920 allogeneic blood products were transfused during the five-year observation period. Of these, 2374 were RBC (60.6%), 148 platelets (3.7%) and 1398 FFP (35.7%) units. Over the entire hospitalization, transfused OMFS patients received a mean of 3.5 (± 4.8) RBC units. Stratified by timing, 42.4% of transfused patients received a mean of 2.1 (± 1.5) RBC units intraoperatively (see Table 1). 3.5% of transfused patients received RBC transfusions preoperatively (1.7 ± 0.5 units) and 54.1% postoperatively (2.1 ± 1.7 units). FFP transfusions were administered in 15.1% of transfused patients intraoperatively (1.4 ± 0.7 units), compared with 0.3% preoperatively (2.5 ± 0.7 units) and 20.6% postoperatively (1.6 ± 0.8 units).

**Table 1:** Patient demographic and clinical characteristics in dependence of transfusion

Characteristic	*All patients*	*Transfusion*		
	*n* (%)	No	Yes	*p* ^1^
Number of patients	13,239	12,570	669	
Age - years				< 0.001
Mean (± SD)	49.1 (± 2.2)	48.3 (± 24.2)	64.3 (± 16.2)	
Sex				0.9
Female	5473 (41.3%)	5198 (41.4%)	275 (41.1%)	
Male	7766 (58.7%)	7372 (58.6%)	394 (58.9%)	
Obesity				0.7
Not obese	12,644 (95.5%)	12,001 (95.5%)	643 (96.1%)	
Grade 1	317 (2.4%)	304 (2.4%)	13 (1.9%)	
Grade 2	156 (1.2%)	150 (1.2%)	6 (0.9%)	
Grade 3	122 (0.9%)	115 (0.9%)	7 (1.0%)	
RBC units				< 0.001
Overall, mean (± SD)	0.2 (± 1.3)	0.0 (± 0.0)	3.5 (± 4.8)	
Intraoperative, mean (± SD)	0.0 (± 0.3)	0.0 (± 0.0)	2.1 (± 1.5) ^+^	
Platelet units				< 0.001
Overall, mean (± SD)	0.0 (± 0.2)	0.0 (± 0.0)	0.2 (± 1.1)	
Intraoperative, mean (± SD)	0.0 (± 0.1)	0.0 (± 0.0)	2.2 (± 1.4) ^+^	
FFP units				< 0.001
Overall, mean (± SD)	0.1 (± 1.2)	0.0 (± 0.0)	2.1 (± 4.9)	
Intraoperative, mean (± SD)	0.0 (± 0.3)	0.0 (± 0.0)	1.4 (± 0.7) ^+^	
Preoperative hemoglobin - g/dl				< 0.001
Mean (± SD)	13.5 (± 1.9)	13.6 (± 1.8)	11.9 (± 2.4)	
Postoperative hemoglobin - g/dl				< 0.001
Mean (± SD)	11.3 (± 2.1)	11.8 (± 1.9)	9.0 (± 1.4)	
Hemoglobin difference pre- to postoperative				< 0.001
Mean (± SD)	−1.8 (± 1.8)	−1.5 (± 1.5)	−3.0 (± 2.2)	
Preoperative anemia*				< 0.001
No	7772 (70.8%)	7494 (72.7%)	278 (41.9%)	
Yes	3204 (29.2%)	2818 (27.3%)	386 (58.1%)	
Postoperative anemia*				< 0.001
No	887 (25.2%)	879 (30.6%)	8 (1.2%)	
Yes	2634 (74.8%)	1991 (69.4%)	643 (98.8%)	
Preoperative hemoglobin < 7 g/dl*				< 0.001
No	10,948 (99.7%)	10,304 (99.9%)	644 (97.0%)	
Yes	28 (0.3%)	8 (0.1%)	20 (3.0%)	
Postoperative hemoglobin < 7 g/dl*				< 0.001
No	3459 (98.2%)	2855 (99.5%)	604 (92.8%)	
Yes	62 (1.8%)	15 (0.5%)	47 (7.2%)	
Preoperative hemoglobin < 9 g/dl*				< 0.001
No	10,742 (97.9%)	10,171 (98.6%)	571 (86.0%)	
Yes	234 (2.1%)	141 (1.4%)	93 (14.0%)	
Postoperative hemoglobin < 9 g/dl*				< 0.001
No	2992 (85.0%)	2682 (93.4%)	310 (47.6%)	
Yes	529 (15.0%)	188 (6.6%)	341 (52.4%)	
Surgery duration – minutes				< 0.001
Mean (± SD)	89.3 (± 129.1)	71.3 (± 89.1)	313.1 (± 266.7)	
Diagnoses				
Malignant neoplasms	2606 (19.7%)	2136 (17.0%)	470 (70.3%)	< 0.001
Benign neoplasms	581 (4.4%)	573 (4.6%)	8 (1.2%)	< 0.001
Diseases of the respiratory system	312 (2.4%)	310 (2.5%)	2 (0.3%)	< 0.001
Diseases of the oral cavity, salivary glands and jaws	5953 (45.0%)	5828 (46.4%)	125 (18.7%)	< 0.001
Diseases of the skin and subcutaneous tissue	114 (0.9%)	112 (0.9%)	2 (0.3%)	< 0.001
Diseases of the musculoskeletal system and connective tissue	111 (0.8%)	108 (0.9%)	3 (0.4%)	< 0.001
Congenital malformations of eye, ear, face and neck	406 (3.1%)	398 (3.2%)	8 (1.2%)	< 0.001
Trauma	2908 (22.0%)	2876 (22.9%)	32 (4.8%)	< 0.001
Complications of surgical and medical treatment	151 (1.1%)	133 (1.1%)	18 (2.7%)	< 0.001
Other OMFS diagnoses	97 (0.7%)	96 (0.8%)	1 (0.1%)	< 0.001

^1^Wilcoxon rank sum test, t-test, Pearson Chi-square test, Fisher’s exact test

*As far as hemoglobin was recorded (numbers do not equal 13239 due to missing hemoglobin data, as measurements were not performed in all cases)

^+^Intraoperative mean (± SD) refers to patients receiving the respective blood product intraoperatively

(*SD* = standard deviation; *RBC* = red blood cell; *FFP* = fresh frozen plasma; *OMFS* = Oral and maxillofacial surgery; *overall* = entire time of hospitalization; *intraoperative* = during surgery)

Platelet transfusions were given in 2.1% of transfused patients intraoperatively (2.2 ± 1.4 units), 1.8% preoperatively (2.0 ± 1.2 units), and 0.7% postoperatively (2.2 ± 1.1 units). Autologous transfusion procedures such as cell salvage were not used. 29.2% (*n* = 3204) of the patients with recorded preoperative hemoglobin had preoperative anemia. The proportion of transfused patients with preoperative anemia (58.1%; *n* = 386) was significantly higher (*p* < 0.001) than in non-transfused patients (27.3%; *n* = 2818). There was also a significantly (*p* < 0.001) higher proportion of postoperative anemia in transfused (98.8%; *n* = 643) than in non-transfused (69.4%; *n* = 1991) patients.

In the transfused patient cohort, the group of malignant neoplasms was most strongly represented with a total of 470 patients (70.3%) (see Table 1).

Patients were divided into those with and without preoperative anemia. In presence of preoperative anemia, significant differences were identified between patients regarding ICU treatment, in-hospital mortality and H-LOS (*p* < 0.001). Patients with preoperative anemia were significantly more likely to receive ICU treatment than patients without preoperative anemia (16.1% vs. 9.2%; *p* < 0.001). In-hospital mortality was significantly higher with diagnosed preoperative anemia than without (1.0% vs. 0.2%; *p* < 0.001). The average H-LOS for patients with preoperative anemia was 6.7 (± 10.0) days compared to an inpatient stay of 4.3 (± 6.7) days for patients without anemia (*p* < 0.001) (see Table 2).

**Table 2:** Transfusions and course of treatment in patients with versus without preoperative anemia

Characteristics	*All patients*	*Preoperative anemia*		
	*n* (%)	No	Yes	*p* ^1^
Number of patients*	10,976	7773	3204	
Allogeneic blood units, overall				< 0.001
Mean (± SD)	0.4 (± 2.7)	0.2 (± 2.4)	0.7 (± 3.3)	
RBC units, overall				< 0.001
Mean (± SD)	0.2 (± 1.5)	0.1 (± 1.2)	0.5 (± 2.0)	
Platelet units, overall				< 0.001
Mean (± SD)	0.0 (± 0.3)	0.0 (± 0.2)	0.0 (± 0.4)	
FFP units, overall				< 0.001
Mean (± SD)	0.1 (± 1.3)	0.1 (± 1.2)	0.2 (± 1.5)	
ICU treatment				< 0.001
Yes	1233 (11.2%)	717 (9.2%)	516 (16.1%)	
No	9743 (88.8%)	7055 (90.8%)	2688 (83.9%)	
In-hospital mortality				< 0.001
Yes	46 (0.4%)	15 (0.2%)	31 (1.0%)	
No	10,930 (99.6%)	7757 (99.8%)	3173 (83.9%)	
H-LOS – days				< 0.001
Mean (± SD)	5.0 (± 7.6)	4.3 (± 6.1)	6.7 (± 10.0)	

^1^Wilcoxon rank-sum test, t-test, Pearson chi-square test

*As far as hemoglobin was recorded (numbers do not equal 13239 due to missing hemoglobin data, as measurements were not performed in all cases)

(*SD* = standard deviation; *RBC* = red blood cell; *FFP* = fresh frozen plasma; *ICU* = intensive care unit; *H-LOS* = hospital length of stay)

The overall transfusion rate of OMFS diagnoses was 5.1% (*n* = 669/13,239; 95% CI 4.7–5.4). The diagnosis group with the highest transfusion rate of 18.0% consisted of all patients with malignant neoplasms, regardless of the type or extent of their surgical treatment (see Table 3).

**Table 3:** Transfusion rates

	*All patients*	*Transfusion*		*Transfusion rate*	
	*n*	No	Yes	%	95% CI
Diagnoses					
Malignant neoplasms	2606	2136	470	18.0%	16.6–19.6
Benign neoplasms	581	573	8	1.4%	0.7–2.7
Diseases of the respiratory system	312	310	2	0.6%	0.2–2.3
Diseases of the oral cavity, salivary glands and jaws	5953	5828	125	2.1%	1.8–2.5
Diseases of the skin and subcutaneous tissue	114	112	2	1.8%	0.5–6.2
Diseases of the musculoskeletal system and connective tissue	111	108	3	2.7%	0.9–7.6
Congenital malformations of eye, ear, face and neck	406	398	8	2.0%	1.0–3.8
Trauma	2908	2876	32	1.1%	0.8–1.5
Complications of surgical and medical treatment	151	133	18	11.9%	7.7–18.1
Other OMFS diagnoses	97	96	1	1.0%	0.1–5.6

(Transfusion rate (%) = number of transfused patients/number of all patients; OMFS = Oral and maxillofacial surgery)

Increased transfusion rates were also found for surgical complications (11.9%) and craniofacial deformities (26.3%; *n* = 5/19; 95% CI 11.8–48.8), such as craniosynostosis. Osteomyelitis, osteoradionecrosis (ORN) and medication-related osteonecrosis of the jaw (MRONJ) had a transfusion rate of 7.1% (*n* = 80/1122; 95% CI 5.8–8.8), independent of type or extent of surgery.

OMFS pathologies that did not have an elevated transfusion rate included clefts (0.0%; *n* = 0/328; 95% CI 0.0–1.2) and dysgnathia with a transfusion rate of 0.6% (*n* = 4/669; 95% CI 0.2–1.5).

Owing to the high transfusion rate in the group of malignant neoplasms, further analyses were performed, including a focused assessment on free flap reconstruction surgeries. Table 4 provides an overview of transfusion requirements across free flap types and for different pathologies. In total 58.8% of 796 free flap patients required transfusions. Surgeries, where patients were reconstructed with scapula (87.5%), anterolateral thigh (79.7%), and fibula free flaps (78.9%) showed high transfusion rates. Radial forearm free flaps demonstrated the lowest rate (34.3%), while latissimus dorsi (71.0%) and deep circumflex iliac artery free flaps (43.8%) showed intermediate frequencies. Simultaneous free flaps exhibited the highest rate in all free flap surgeries (91.7%).

**Table 4:** Subgroup analysis of transfusion rates for free flap surgery

	*All patients*	*Transfusion*		*Transfusion rate*	
	*n*	No	Yes	%	95% CI
Overall free flaps	796	328	468	58.8%	55.3–62.2
Free flap type					
Fibula	275	58	217	78.9%	73.5–83.5
Scapula	40	5	35	87.5%	72.4–95.3
Radial forearm	347	228	119	34.3%	29.5–39.4
Anterolateral thigh	69	14	55	79.7%	68.0–88.1
Latissimus dorsi	31	9	22	71.0%	51.8–85.1
Deep circumflex iliac artery	16	9	7	43.8%	20.8–69.4
Other	6	4	2	33.3%	6.0–75.9
Simultaneous	12	1	11	91.7%	59.8–99.6
Diseases of the oral cavity, salivary glands and jaws					
With free flap	84	20	64	76.2%	66.1–84.0
ORN with free flap	63	13	50	79.4%	67.8–87.5
MRONJ with free flap	13	4	9	69.2%	38.9–89.6
Osteomyelitis with free flap	8	3	5	62.5%	25.9–89.8
Without free flap	5869	5808	61	1.0%	0.8–1.3
Trauma					
With free flap	6	4	2	33.3%	6.0–75.9
Without free flap	2902	2872	30	1.0%	0.7–1.5
Malignant neoplasms					
With free flap	669	278	391	58.4%	54.6–62.2
OSCC with free flap	566	228	338	59.7%	55.6–63.6
Other than OSCC with free flap	103	50	53	51.5%	41.9–60.9
Without free flap	1937	1858	79	4.1%	3.3–5.1
Other OMFS with free flap	37	21	16	43.2%	27.5–60.4

(Transfusion rate (%) = number of transfused patients/number of all patients; OMFS = Oral and maxillofacial surgery; OSCC= oral squamous cell carcinoma; ORN = osteoradionecrosis; MRONJ = medication related osteonecrosis of the jaw)

Diseases of the oral cavity, salivary glands and jaws treated with reconstructive free flap surgery had a transfusion rate of 76.2%, compared with 1.0% among patients treated without free flap surgery in this group. Within this group, ORN (79.4%), MRONJ (69.2%), and osteomyelitis (62.5%) constituted the relevant subcategories. Trauma patients reconstructed with free flaps showed a lower rate (33.3%). For malignant neoplasms 58.4% of patients undergoing free flap surgery required transfusion, compared with 4.1% of those not reconstructed with a free flap (e.g. biopsy, excision, local flap). OSCC patients with free flap reconstruction showed a transfusion rate of 59.7%, while other malignancies demonstrated a rate of (51.5%).

Multivariate regression analyses revealed that female sex (OR 1.54; 95% CI 1.23–1.93; *p* < 0.001), age (OR 1.01; 95% CI 1.01–1.02; *p* < 0.001), preoperative anemia (OR 6.25; 95% CI 4.89–8.04; *p* < 0.001), malignant neoplasm (OR 1.38; 95% CI 1.05–1.82; *p* = 0.022), microvascular free flap surgery (OR 5.21; 95% CI 3.53–7.70; *p* < 0.001) and increased surgery duration (OR 1.01; 95% CI 1.01–1.01; *p* < 0.001) were significant influencing factors on transfusion rates (see Table 5).

**Table 5:** Univariate and multivariate logistic regression analyses of influencing factors on transfusion rate in all OMFS patients

	*Transfusion*		*Univariate analysis*			*Multivariate analysis*		
	*No*	*Yes*						
Variable	n (%)	n (%)	OR	95% CI	p	OR	95% CI	p
Age – years								
Mean (± SD)	48.3 (± 24.2)	64.3 (± 16.2)	1.03	1.03–1.04	< 0.001	1.01	1.01–1.02	< 0.001
Preoperative anemia*								
No	7494 (96.4%)	278 (3.6%)	-	-	-	-	-	-
Yes	2818 (88.0%)	386 (12.0%)	3.69	3.15–4.34	< 0.001	6.25	4.89–8.04	< 0.001
Free flap								
No	12,242 (98.4%)	201 (1.6%)	-	-	-	-	-	-
Yes	328 (41.2%)	468 (58.8%)	86.90	71.39–106.17	< 0.001	5.21	3.53–7.70	< 0.001
Sex								
Male	7372 (94.9%)	394 (5.1%)	-	-	-	-	-	-
Female	5198 (95.0%)	275 (5.0%)	0.99	0.85–1.16	0.900	1.54	1.23–1.93	< 0.001
Obesity								
Not obese	12,001 (94.9%)	643 (5.1%)	-	-	-	-	-	-
Grade 1	304 (95.9%)	13 (4.1%)	0.80	0.43–1.34	0.431	0.39	0.16–0.82	0.020
Grade 2	150 (96.2%)	6 (3.8%)	0.75	0.29–1.55	0.485	0.47	0.15–1.33	0.185
Grade 3	115 (94.3%)	7 (5.7%)	1.14	0.48–2.27	0.744	1.32	0.48–3.21	0.569
Surgery duration – minutes								
Mean (± SD)	72.2 (± 89.3)	422.1 (± 252.5)	1.01	1.01–1.01	< 0.001	1.01	1.01–1.01	< 0.001
Malignant neoplasm								
No	10,434 (98.1%)	199 (1.9%)	-	-	-	-	-	-
Yes	2136 (82.0%)	470 (18.0%)	11.54	9.73–13.73	< 0.001	1.38	1.05–1.82	0.022

*As far as hemoglobin was recorded (numbers do not equal 13239 due to missing hemoglobin data, as measurements were not performed in all cases)

**Fig. 1:**
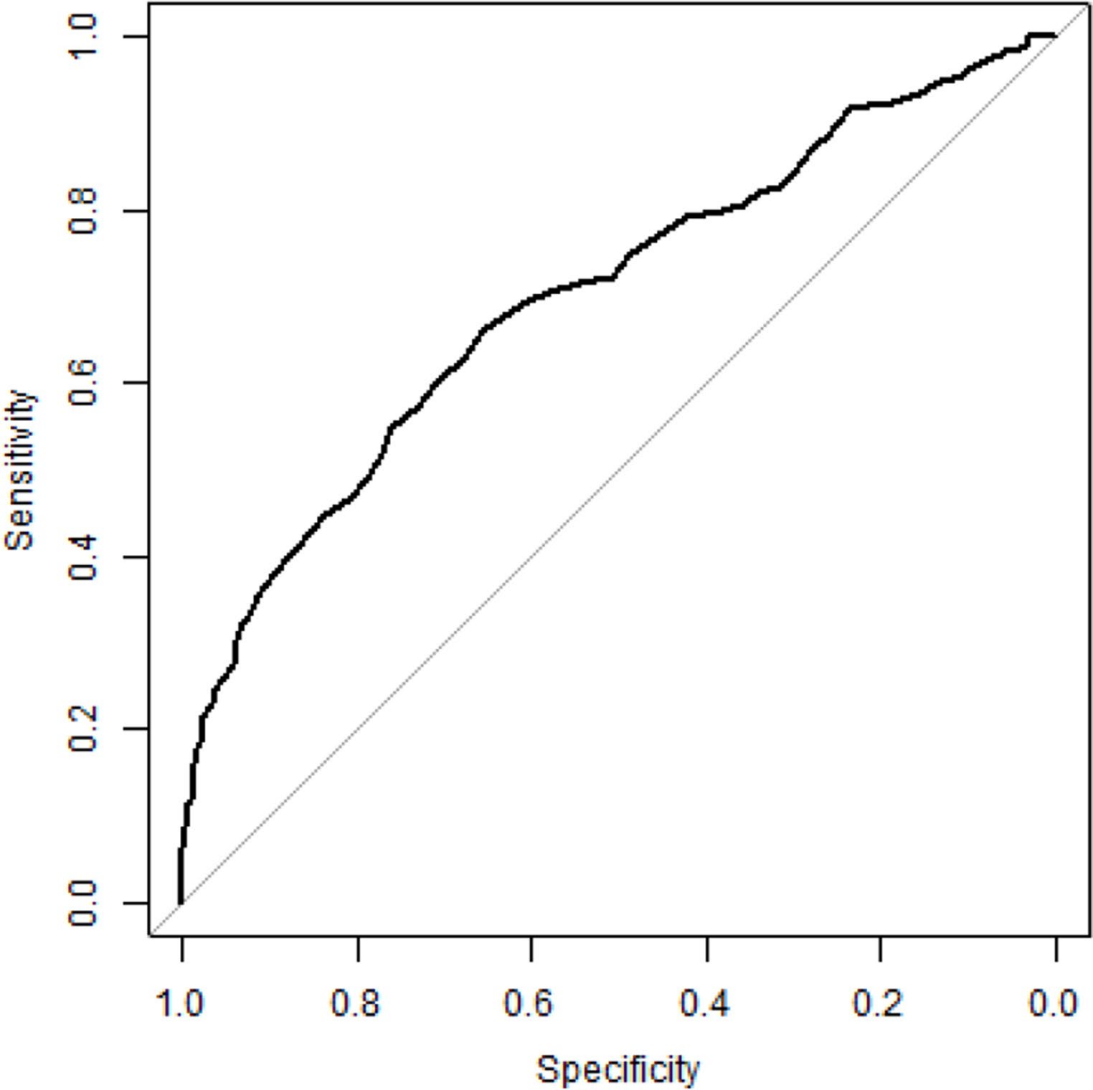
ROC curve of preoperative hemoglobin for overall transfusion (AUC = 0.70). The diagonal line indicates no discrimination

**Fig. 2:**
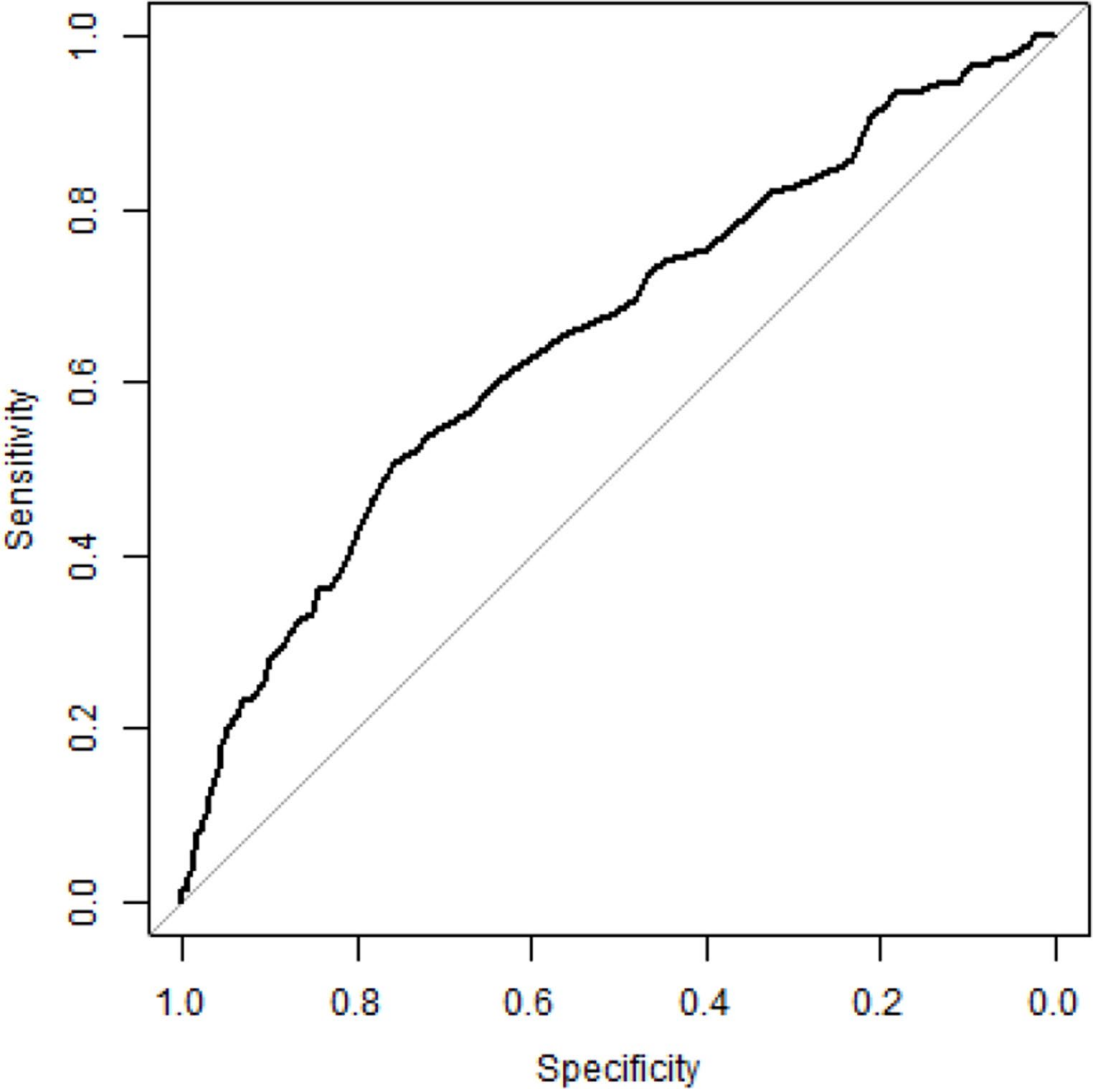
ROC curve of preoperative hemoglobin for intraoperative transfusion (AUC = 0.66). The diagonal line indicates no discrimination

In patients undergoing reconstructive free flap surgery ROC analysis of preoperative hemoglobin levels showed an AUC of 0.70 for overall transfusion and 0.66 for intraoperative transfusion. ROC curves are shown in Figs. 1 and 2.

The hemoglobin cut-off values identified by the Youden index were 13.25 g/dl for overall transfusion risk and 12.25 g/dl for intraoperative transfusion risk. Corresponding sensitivity and specificity values are presented in Table 6. Predictive values (PPV and NPV) for overall and intraoperative transfusion are also summarized in Table 6.

**Table 6:** ROC analysis of preoperative hemoglobin for overall and intraoperative transfusion

	*AUC*	*Hemoglobin threshold (g/dl)* ^*1*^	*Sensitivity (%)*	*Specificity (%)*	*PPV (%)*	*NPV (%)*
Overall transfusion	0.70	13.25	66.0	65.5	73.2	57.5
Intraoperative transfusion	0.66	12.25	50.7	75.9	32.8	86.9

^1^Youden Index

(AUC = area under the curve; PPV = positive predictive value; NPV = negative predictive value; overall = entire time of hospitalization; intraoperative = during surgery)

## Discussion

This study provides a first and uniquely comprehensive overview of transfusion rates across the full spectrum of diagnoses encountered in OMFS. Existing literature has predominantly focused on subgroups - such as free flap reconstruction [7, 9, 14, 18], OSCC [5, 15], orthognathic surgery [10, 11], craniofacial deformities [12, 13] or cleft repair [19, 20] resulting in limited comparability and a lack of integrated evidence across diagnostic categories. In contrast, the present analysis spans a five-year period and includes more than 13,000 operated patients, making it a representative and confirmatory dataset to systematically advocate recommendations towards optimal transfusion requirements in this surgical field.

Across all diagnoses the overall transfusion rate was 5.1%, indicating that routine PBM measures are not required for most OMFS pathologies. However, malignant neoplasms showed an elevated transfusion rate of overall 18.0%. The comparatively lower rate observed in our total cancer cohort, reflects the broad diagnostic grouping prior to stratification by reconstructive technique. Once procedure-specific analysis for free flap reconstruction was applied, increased transfusion probabilities aligned with previously published data. Microvascular free flap surgeries demonstrated increased transfusion requirements, with an overall transfusion rate of 58.8%. This is consistent with studies reporting high transfusion rates in major free flap surgery [7, 9, 14, 15, 18]. In our cohort, simultaneous free-flap reconstructions - performed for complex head and neck defects - showed the highest transfusion rate of 91.7%. This finding is consistent with the substantial operative complexity and physiological demands described for simultaneous free-flap reconstruction in extensive maxillofacial defects, as outlined by Fenske et al. [21], which provide a plausible explanation for the markedly elevated transfusion requirements in these cases.

Importantly, our data show that elevated transfusion needs extend beyond oncologic indications. Conditions such as ORN, MRONJ and advanced osteomyelitis showed high transfusion probabilities similar to oncologic indications when free flap reconstruction was required, whereas the same diagnoses treated without free flaps demonstrated transfusion rates < 5%. This highlights that free flap surgery is the primary determinant of transfusion need, irrespective of the underlying pathology. Perioperative blood loss in patients undergoing microvascular reconstruction reflects both the underlying surgical defect and the flap procedure itself, and cannot clearly be attributed to either component alone, as resection and reconstruction are usually performed as single-stage surgery in our institution.

In addition to malignant neoplasms and free flap procedures, craniofacial deformities showed a high transfusion rate. The elevated rate in craniofacial deformities is consistent with reports by Suarez et al. [13]. It differs from the transfusion rate described by Daly et al. (56%) [22], with our cohort exhibiting a lower rate of 26.3%. This discrepancy is likely influenced by the small number of patients treated operatively in our center, as many craniofacial cases are managed by pediatric neurosurgery as the supervising department. Nevertheless, the transfusion rate observed in our study still reflects the relevant perioperative bleeding risk associated with these surgeries and should be acknowledged in perioperative planning.

Furthermore, several diagnoses showed very low transfusion rates in this large cohort such as clefts, dysgnathia and trauma. These findings support the notion that routine PBM measures are less important in these patient groups.

Across the entire cohort, preoperative anemia, malignant neoplasm and free flap surgery were identified as relevant predictors of transfusion, consistent with prior evidence [5, 15, 18, 23]. Beyond its impact on transfusion probability, preoperative anemia was also associated with a less favorable course of treatment, including higher rates of ICU treatment, increased in-hospital mortality, and prolonged H-LOS in the full cohort, underscoring the importance of early anemia detection and correction as a central component of PBM in OMFS. Previous publications also point in this direction, suggesting that preoperative anemia is an independent risk factor for postoperative complications and associated with an increase in morbidity and mortality [1, 7, 24–27].

Besides these factors confirmed in our analysis, previous studies have demonstrated that patient-related variables - such as age, sex, body weight and tumor stage in malignant neoplasms - can influence transfusion requirements. Moreover, several intraoperative characteristics, including the duration of surgery and the use of microvascular free flap reconstruction, have been identified as contributors to increased transfusion likelihood [4, 5, 11, 15, 18, 23, 28].

Preoperative hemoglobin remains the most widely recognized predictor of transfusion [4, 5, 7, 11, 15, 23, 26, 29] and our data reinforce the central role of anemia as a modifiable risk factor within PBM strategies in OMFS. Thus, our results, derived from the largest OMFS cohort analyzed to date, confirm and strengthen these associations previously reported in smaller studies and thereby provide robust evidence for their relevance in routine clinical practice.

In this context, further evaluation using ROC analysis in the free flap surgery subgroup identified hemoglobin thresholds of 13.25 g/dl for overall transfusion risk and 12.25 g/dl for intraoperative transfusion risk. These findings suggest that even hemoglobin levels close to anemia thresholds or mild preoperative anemia may increase transfusion likelihood in this OMFS subgroup and may therefore serve as clinically relevant indicator for risk-adapted perioperative planning.

These findings are in line with recent evidence in head and neck oncologic surgery. Ali et al. [29] reported a marked increase in transfusion risk and blood product utilization below a hemoglobin threshold of 12.0 g/dl, with preoperative hemoglobin emerging as the strongest predictor of transfusion. While their analysis focused exclusively on oncologic patients, the comparable magnitude of risk observed in our free flap cohort, irrespective of malignant or benign pathology, supports the general relevance of hemoglobin-based risk stratification in major OMFS reconstructive surgery.

Importantly, the identified hemoglobin thresholds should not be interpreted as transfusion triggers. Rather, they represent preoperative risk markers, indicating the need for early anemia detection and therapy. While transfusion triggers remain substantially lower and are also guided by intraoperative and clinical parameters [3], the present findings support a shift towards earlier identification and treatment of anemia to reduce transfusion rates.

The strength of this study lies in its large sample size, long observation period, and the systematic capture of transfusion rates across all diagnoses treated within a single center. This overview fills an important evidence gap, offering transfusion probabilities across the full range of OMFS.

A potential limitation, however, is that the analysis was differentiated by diagnosis rather than by specific surgical procedures, which may influence the comparability of transfusion rates. Nevertheless, this diagnostic-based approach was chosen deliberately to provide a broad and clinically meaningful overview of transfusion requirements across the major fields of OMFS. Additionally, given the particularly high transfusion rates observed in certain diagnostic categories, a targeted subgroup analysis of microvascular free flap reconstructions was conducted to further delineate surgery-specific transfusion requirements. Another limitation of the present study was the retrospective design with potentially missing data, e.g. hemoglobin values. Furthermore, it is possible that factors may have led to transfusions that were not registered in this study. Additionally, intraoperative blood loss data was not available for analysis, which may confound transfusion risk.

At our institution, RBC transfusion practice follows a moderately restrictive approach, typically applying a hemoglobin threshold < 8 g/dl in major OMFS reconstructive surgeries. This threshold is higher than the < 7 g/dl threshold recommended for most surgical patients in national guidelines [3]. This transfusion practice may have influenced the transfusion rates observed in the present cohort and should be considered when interpreting the results.

In major free flap reconstructive OMFS surgeries of oncologic patients, current evidence supports intravenous iron therapy as a safe and effective option to improve hemoglobin levels within a limited preoperative time window, whereas oral iron supplementation and erythropoiesis-stimulating agents may be less effective or require cautious use due to limited efficacy, delayed onset, or potential safety concerns [29]. Nevertheless, in non-oncologic patients with iron deficiency oral iron supplementation is a feasible approach when sufficient time is available prior to surgery.

Beyond preoperative optimization, intraoperative strategies may further contribute to reducing transfusion requirements. A range of adjunctive techniques and hemostatic interventions, including electrocautery, ultrasonic devices, tranexamic acid and maintenance of normovolemia, have been proposed to minimize perioperative blood loss and, consequently, the need for transfusion [29].

Studies outside OMFS have demonstrated that structured PBM programs can reduce transfusion requirements and improve clinical outcomes [30–32]. Prospective multicenter studies could help validate these findings and further clarify procedure-specific transfusion risks. Nevertheless, the present data provide an essential foundation for evidence-based PBM planning in OMFS, including the prioritization of anemia screening and treatment to optimize preoperative hemoglobin levels and targeted preparation of blood products to improve perioperative hemotherapy.

## Conclusion

From a clinical perspective for PBM guidance in OMFS the following recommendations can be derived:

1. Routine preoperative anemia screening should be performed sufficiently in advance in OMFS patients, particularly prior to major procedures such as microvascular free flap surgery, to enable timely diagnostic clarification and integration of guideline-based therapy within PBM pathways.
2. Preoperative anemia should be managed with a cause-directed approach whenever feasible. In patients undergoing high-risk OMFS procedures such as microvascular free flap surgery, intravenous iron therapy should be considered as a primary treatment option, in particular when time to surgery is limited. Oral iron supplementation may be appropriate in selected patients with sufficient time prior to surgery.
3. High-risk OMFS surgeries, especially microvascular free flap reconstruction, require intensified PBM measures, including early anemia workup. In these patients decreased preoperative hemoglobin should be noticed as a risk marker indicating an increased likelihood of transfusion requirement and the need for transfusion rate-oriented preparation of blood products to optimize perioperative management.

## Supplementary Information

Below is the link to the electronic supplementary material.

ESM 1 (DOCX 13.2 KB)

## Data Availability

Data is provided within the manuscript. Raw data cannot be shared to protect study participant privacy as direct patient data were involved in the analysis.

## Declarations

During the preparation of this work the authors used Chat GPT-5.1 architecture to improve language style, text clarity and translation. The tool was not employed for data analysis, interpretation, or any task that could influence the study’s outcome or conclusions. After using this tool, the authors carefully reviewed and edited the content as required and take full responsibility for the content of the publication.
