# Supplementary material for: Transfusion rates in oral and maxillofacial surgery and their influencing factors in the context of patient blood management
Source: Clin Oral Investig. 2026 May 13;30(6):231. doi: 10.1007/s00784-026-06902-y (PMC13171644; doi:10.1007/s00784-026-06902-y)
Supplement: Supplementary file 1 — (DOCX 13.2 KB) [file 784_2026_6902_MOESM1_ESM.docx]

**Supplementary Table:** ICD-10-GM codes of oral and maxillofacial surgery diagnoses included in the analysis

| Diagnoses | ICD-10-GM Codes |
| --- | --- |
| Malignant neoplasms | C00, C01, C02, C03, C04, C05, C06, C07, C08, C10, C31, C41, C43, C44, C49, C69, C77, C79 |
| Benign neoplasms | D10, D11, D16, D17, D18, D21, D23, D36 |
| Diseases of the respiratory system | J01, J32, J34, J95 |
| Diseases of the oral cavity, salivary glands and jaws | K00, K01, K02, K04, K05, K07, K08, K09, K10, K11, K12, K13, K14 |
| Diseases of the skin and subcutaneous tissue | L02, L03, L08, L72, L90, L91 |
| Diseases of the musculoskeletal system and connective tissue | M12, M24, M72, M84, M86, M87, M95 |
| Congenital malformations of eye, ear, face and neck | Q10, Q17, Q18, Q30, Q35, Q36, Q37, Q38, Q67, Q75 |
| Trauma | S00, S01, S02, S03, S04, S05, S06, S08, S09, S10, S11, S14, S15, S62, S92 |
| Complications of surgical and medical treatment | T81, T84, T85, T86 |
| Other OMFS diagnoses | G47, G50, G51, G52, H02, H05, R02, R59 |
